# Effects of combined drug treatments on *Plasmodium falciparum*: *In vitro* assays with doxycycline, ivermectin and efflux pump inhibitors

**DOI:** 10.1371/journal.pone.0232171

**Published:** 2020-04-23

**Authors:** Riccardo Nodari, Yolanda Corbett, Ilaria Varotto-Boccazzi, Daniele Porretta, Donatella Taramelli, Sara Epis, Claudio Bandi

**Affiliations:** 1 Department of Biosciences and Pediatric Clinical Research Center Romeo ed Enrica Invernizzi, University of Milan, Milan, Italy; 2 Centro Interuniversitario di Ricerca sulla Malaria/Italian Malaria Network, Milan, Italy; 3 Department of Environmental Biology, Sapienza University of Rome, Rome, Italy; 4 Department of Pharmacological and Biomolecular Sciences, University of Milan, Milan, Italy; Instituto Rene Rachou, BRAZIL

## Abstract

There is great concern regarding the rapid emergence and spread of drug-resistance in *Plasmodium falciparum*, the parasite responsible for the most severe form of human malaria. Parasite populations resistant to some or all the currently available antimalarial treatments are present in different world regions. Considering the need for novel and integrated approaches to control malaria, combinations of drugs were tested on *P*. *falciparum*. The primary focus was on doxycycline, an antibiotic that specifically targets the apicoplast of the parasite. In combination with doxycycline, three different drugs known to inhibit efflux pumps (verapamil, elacridar and ivermectin) were tested, with the assumption that they could increase the intracellular concentration of the antibiotic and consequently its efficacy against *P*. *falciparum*. We emphasize that elacridar is a third-generation ABC transporters inhibitor, never tested before on malaria parasites. *In vitro* experiments were performed on asexual stages of two strains of *P*. *falciparum*, chloroquine-sensitive (D10) and chloroquine-resistant (W2). Incubation times on asynchronous or synchronous cultures were 72h or 96h, respectively. The antiplasmodial effect (*i*.*e*. the IC_50_) was determined by measuring the activity of the parasite lactate dehydrogenase, while the interaction between drugs was determined through combination index (CI) analyses. Elacridar achieved an IC_50_ concentration comparable to that of ivermectin, approx. 10-fold lower than that of verapamil, the other tested ABC transporter inhibitor. CI results showed synergistic effect of verapamil plus doxycycline, which is coherent with the starting hypothesis, *i*.*e*. that ABC transporters represent potential targets, worth of further investigations, towards the development of companion molecules useful to enhance the efficacy of antimalarial drugs. At the same time, the observed antagonistic effect of doxycycline in combination with ivermectin or elacridar highlighted the importance of drug testing, to avoid the *de-facto* generation of a sub-dosage, a condition that facilitates the development of drug resistance.

## Introduction

Despite the efforts to eradicate malaria, the disease is still one of the major causes of death in low-income countries, with over 200 million cases in 2018, and about half a million deaths. In the last few years, the trend of malaria decrease has levelled off, with a slight although worrisome increase in malaria cases in the period 2015–2017 [1]. This recent increase in malaria incidence can be attributed to many social and economic factors, but also to the development of multiple resistances of the parasites to antimalarial drugs [2, 3], and of the vectors towards insecticides [4, 5]. *Plasmodium falciparum* has developed resistance to all the currently available antimalarial drugs [6]. The factors that influence the emergence and spread of resistance are not completely known, but drug pressure is likely a key element for the selection of resistant parasite mutants [7].

Combined drug therapies, *i*.*e*. the association of different drugs with different mechanisms of action and pharmacokinetics, are proving to be more effective for malaria control than monotherapy [8] and have been introduced also with the intent to delay the onset of resistance. Since 2001, WHO recommends artemisinin-based combination therapies (ACTs) as first-line intervention in almost all countries where malaria is endemic [9], but resistance to ACT is also emerging [10]. Thus, the armamentarium of effective antimalarials is quite limited, and the search for novel companion drugs to be used in antimalarial combination therapy is a critical need.

In the area of malaria research, the apicoplast has attracted a great deal of attention, also for its potential as a target for novel therapeutic molecules [11]. The apicoplast is a cellular organelle, present in Apicomplexa parasites, including *Plasmodium* spp. It is considered as a relic of a chloroplast, with no photosynthetic ability [12], but indispensable for the survival of the parasite [13]. The prokaryotic-like metabolic pathways of the apicoplast make it susceptible to several antibacterial drugs [14]. Doxycycline has been shown to specifically target the apicoplast in *Plasmodium* spp., determining an anti-parasite effect [15]. The antimalarial effects of doxycycline are however limited, mainly due to its delayed effect, and the combinations with other drugs are recommended for the use of this antibiotic in malaria control [16].

A strategy that has been proposed to increase the efficacy of antibiotic treatments targeting cellular organelles, intracellular symbionts or pathogenic bacteria is to combine antibiotics with inhibitors of drug efflux pumps [17], such as the ABC transporters. The inhibition of these efflux pumps is expected to increase drug concentration into target cells, organelles and intracellular bacteria [18]. Verapamil is a first generation, widely investigated inhibitor of ABC efflux pumps. Verapamil has already been tested on *P*. *falciparum* [19, 20], but not with the drug combination tested here. Elacridar (GF120918) is a potent, third-generation ABC transporters inhibitor, with a reported ability in blocking efflux pumps of different cell lines *in vitro*, 100-fold more potent than verapamil (*i*.*e*. with the same efficacy of verapamil at 100-fold lower concentrations) [21, 22]. There are no published papers on the effect of elacridar on malaria parasites. A study has however been published on another apicomplexan, *Toxoplasma gondii*, where elacridar was shown to severely inhibit parasite cell invasion and multiplication [23].

Surprisingly, ivermectin, a macrocyclic lactone widely used in antiparasitic chemotherapy, has also been proposed as an inhibitor of drug efflux pumps [24], and shown to act synergistically with doxycycline in the anti-symbiotic chemotherapy of dog filariases [17]. This drug could thus be investigated as a further potential inhibitor of drug efflux pumps, to be used in combination with doxycycline in antimalarial treatment. On the other hand, doxycycline has been shown to increase the intracellular concentration of macrocyclic lactones, which makes the combination treatment ivermectin-doxycycline worth of further investigation [25]. Ivermectin is used in mass therapy in tropical countries for the control of river blindness, in regions in which both the filarial nematode *Onchocerca volvulus* and malaria are endemic. Recent papers have shown that the feeding of mosquitoes on humans treated with ivermectin causes detrimental effects on *Anopheles* mosquitoes, with a reduction of population densities [26, 27]. Moreover, several studies have shown evidences for a reduction in malaria transmission due to the administration of ivermectin [28]. Thus, it has been recommended an intensification of mass administration of ivermectin, since this could lead not only to the control of onchocerciasis but could also contribute to malaria control, through the reduction of vector populations [29]. However, mass administration of ivermectin in malaria-endemic countries implies that a part of the population is temporally exposed at both ivermectin and antimalarial drugs [30]. Thus, should ivermectin interact in an antagonistic way with one or more antimalarial drugs, this could have a negative impact on the control of both filariasis and malaria, with a reduction in efficacy of the treatments and an increase in resistance development.

Based on the above background information, with the goal of investigating novel combinations of molecules that could be detrimental to malaria parasites, this study was focussed on a molecule that targets the apicoplast, doxycycline, on two inhibitors of ABC efflux pumps, verapamil and elacridar, and on ivermectin, a drug that is both interesting as a potential efflux pump inhibitor, as well as it is at the core of parasite control programmes in tropical medicine. Different combinations of these drugs were tested, with *in vitro* assays on asexual stages of *P*. *falciparum*.

## Materials and methods

### Drugs and reagents

Reagents for parasite maintenance and for the assays were purchased from different suppliers: RPMI 1640 medium (GIBCO BRL), AlbuMax II (Invitrogen), hypoxanthine (Sigma-Aldrich), HEPES and L-glutamine (EuroClone). All drugs (elacridar, verapamil, ivermectin, and doxycycline) were purchased from Sigma-Aldrich, maintained in DMSO and diluted for the tests with the culture medium prior to the experiments.

### Parasites and drug susceptibility testing

All the combinations of drugs were tested against two laboratory adapted strains of *P*. *falciparum*: the *P*. *falciparum* clone D10 (from Papua New Guinea, chloroquine susceptible) originally obtained from Prof P. Smith (Div. Clinical Pharmacology, University of Cape Town, SA) and the *P*. *falciparum* Indochina III/CDC, clone W2 (chloroquine resistant) originally obtained from the Walter Reed Army Institute of Research, Washington, DC. Both parasite strains were maintained in continuous cultures by a modification of the methods of Trager and Jensen [31]. Each culture was maintained at 5% haematocrit (human type A+ red blood cells [RBC]) in RPMI 1640 medium supplemented with 1% AlbuMax II (lipid-rich bovine serum albumin), 0,5 mM hypoxanthine, 20 mM HEPES, and 2 mM L-glutamine, in a standard gas mixture consisting of 94% N_2_, 5% CO_2_, and 1% O_2_ at 37°C. RBC were obtained from the AVIS Comunale Milano (www. https://avismi.it) with the written consent of anonymous healthy donors, following the EU Directive 2004/23/EC, 2002/98/EC and the Directive (Suppl. Ord. "G.U n. 300 del 28 dicembre 2015) from the Italian Ministry of Health, on human cells, tissues, blood and blood components.

Synchronization of the parasite, for the experiments with an incubation time corresponding to the duration of 2 asexual cycles, was performed according to published protocols [32].

Parasite susceptibility testing was done with the colorimetric assay of LDH (lactate dehydrogenase) [33]. All the combinations were tested with incubation times of 72h, on asynchronous cultures of the parasites, and 96h, on synchronized cultures of the parasites (late ring stage, approximately 24h post invasion as suggested by Dahl et al. [15]). For the combination test, serial dilutions of the first drug (usually doxycycline) were done directly in a 96 flat bottom well plate. The starting concentration (for serial dilutions) was assigned so that the 50% inhibitory concentration (IC_50_) of each drug would be in the centre of the plate. After that, selected dilutions of the second drug were added to the first one. Treatments with two combined drugs were performed by keeping one drug at fixed concentration(s) while varying the other (selected drug concentrations are reported in the S2 Text). The cultures of parasites were diluted to a final haematocrit of 1% and a final parasitaemia of 1% (or 0,2% at ring stage for 96h incubation time) and placed in a 96 wells plate with the drugs. Each combination of concentrations of the drugs was plated in duplicate, with a triplicate blank sample (only RBC) and a triplicate of negative control (only parasites). The IC_50_ values were determined for each drug alone and for the drugs in combination, directly by the Synergy4-BioTek reader.

The 96-well plates with the suspensions of the drugs and parasites were incubated at 37°C for 72h or 96h. All the combination experiments were performed three independent times.

For the experiments with 96h incubation times, the culture medium has been changed after 24h for the nutrient needs of the parasites. Assays with medium change at different points (48 or 72h –results not shown) were also performed, but the results were similar to the experiments with a change of the media at 24h of incubation. Thus, for more practical scheduling of the experiments, all the tests were done with the change at 24h of incubation.

### Data analyses

Statistical analysis was performed on the values of IC_50_ to compare the effect of drugs against the two strains of *P*. *falciparum* with the two incubation times. The data were analysed using GraphPad Prism 5 utilizing the analysis of variance (two ways ANOVA) followed by Sidak multiple comparisons test (P-values <0.05 have been considered significant).

The synergism, additivity and antagonism of the combination drug treatment were evaluated using the Chou–Talalay combination index method as implemented in the CompuSyn software (CompuSyn Inc., Paramus, NJ) [34, 35, 36]. Combination index (CI) values were calculated using the equation:

$$\text{CI}={\text{C}}_{\text{A,x}}/{\text{IC}}_{\text{x,A}}\;+\;{\text{C}}_{\text{B,x}}/{\text{IC}}_{\text{x,B}}$$

where IC_x,A_ and C_x,B_ are the concentrations of drug A and drug B used as a single agent to produce a given effect x and C_A,x_ and C_B,x_ are the concentrations of drug A and drug B in the combination to produce that same effect. A Fa-CI plot for each drug combination (Chou-Talalay plots), with CI on y- axis as a function of effect level (f_a_) on the x- axis, were generated and used to interpret drug combination effects [34, 35]. Synergism, additivity or antagonism were classified on the basis of the CI values and represented using the recommended symbols in CompuSyn and CalcuSyn manual [35].

## Results

Three different drugs known to inhibit efflux pumps (ivermectin, verapamil, and elacridar) were tested alone or in combination with doxycycline, with the assumption that this could determine an increased intracellular concentration of the antibiotic, thus an increased, and possibly synergistic, effect against *P*. *falciparum*. In addition, the combination of verapamil and ivermectin was also tested.

All the combinations involving doxycycline have been tested with two different incubation times: 72h, on asynchronous cultures of the parasite, and 96h, on synchronous cultures. These two incubation times allowed to observe the effects of the drugs, alone and in combination, on the parasite viability after one complete asexual cycle (72h assay), or after two complete asexual cycles (96h assay). The latter time point was included to determine the effect of doxycycline on parasite viability, which has been reported to be delayed [15].

**Fig 1:**
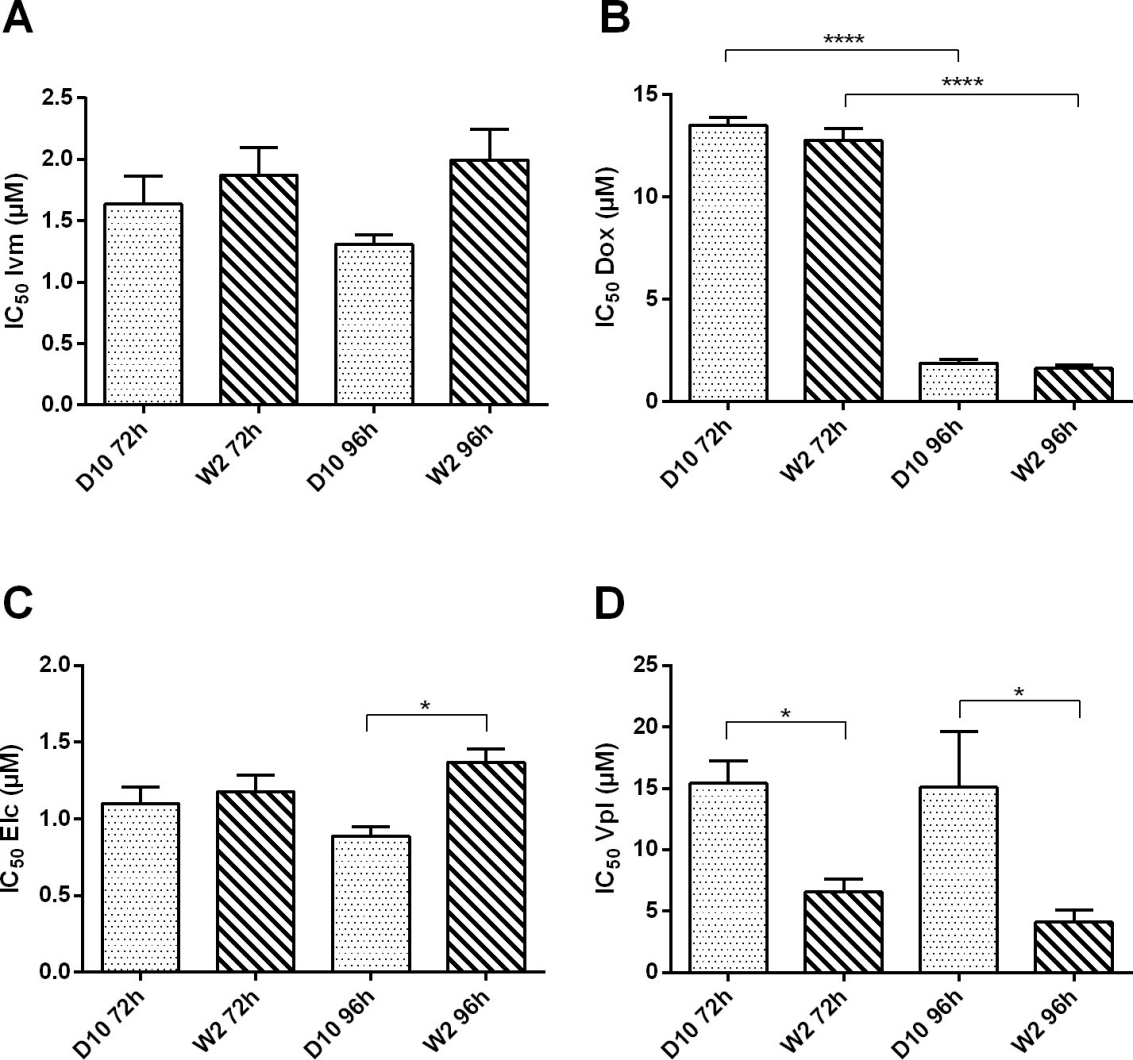
Graphs showing the IC_50_ ± Standard Error of each drug alone on *P*. *falciparum* chloroquine-sensitive (D10) or chloroquine-resistant (W2) strains at 72h or 96h time length bioassays. (A) Ivermectin (Ivm); (B) Doxycycline (Dox); (C) Elacridar (Elc); (D) Verapamil (Vpl). * represents a statistically significative difference between the IC_50_ values (* p<0.05; ** p<0.01; **** p<0.0001).

In a first set of experiments, the IC_50_ of the compounds alone on both D10 and W2 strains of *P*. *falciparum* were determined. The results are shown in Fig 1 and in S1 Table. After 72h of incubation, no significant differences were found between the two strains of *P*. *falciparum* in terms of susceptibility to ivermectin, doxycycline or elacridar (Fig 1A–1C). On the contrary the susceptibility to verapamil was significantly different between the two strains, with a higher effect on the W2 strain with a 72h incubation (p = 0.0233; Fig 1D). After 96h, namely two parasite growth cycles, the susceptibility of both strains to ivermectin did not change significantly (Fig 1A), whereas the IC_50_ of doxycycline decreased more than 7-folds (p < 0.0001 for both strains; Fig 1B). The response to elacridar was similar to that to ivermectin, with the D10 strain being more susceptible than W2 at 96h to both compounds, but the difference was significant only for elacridar (p = 0.0309; Fig 1C). After 96h, verapamil confirmed its strongest activity against W2 than against D10 (p = 0.0254; Fig 1D), an activity that was already evident after 72h.

**Fig 2:**
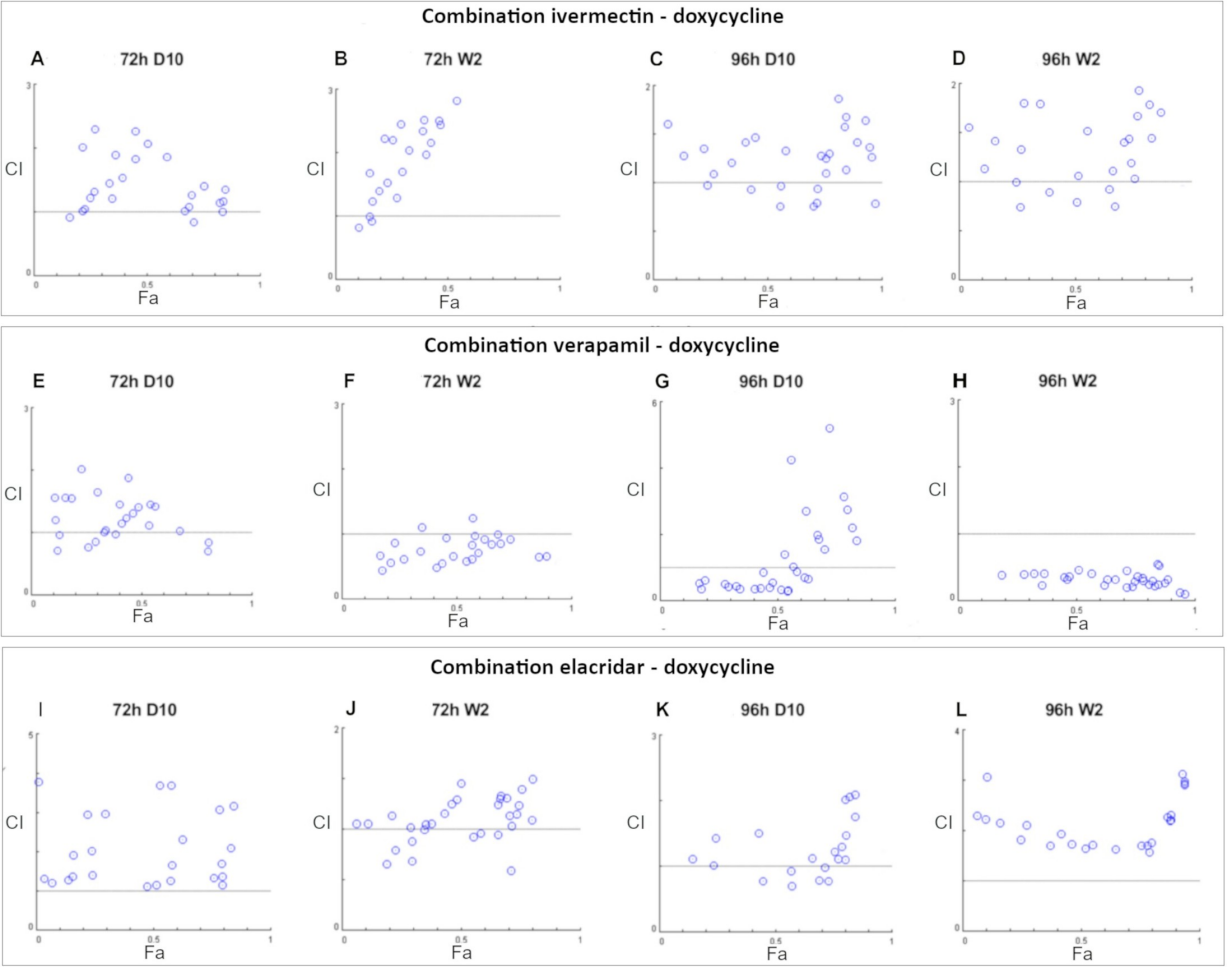
Combination index plots for the anti-*P*. *falciparum* activity of ivermectin (Ivm), verapamil (Vpl) or elacridar (Elc) in combination with doxycycline (Dox) against *P*. *falciparum*. The data are representative of three independent experiments in duplicate for each combination. The 72h assays were performed on asynchronous cultures of *P*. *falciparum*, while the 96h assays were performed on synchronous cultures of the parasites with a medium change after 24h (see M&M for details). Combination of Ivm-Dox (from A to D); combination of Vpl-Dox (from E to H); combination of Elc-Dox (From I to L).

**Fig 3:**
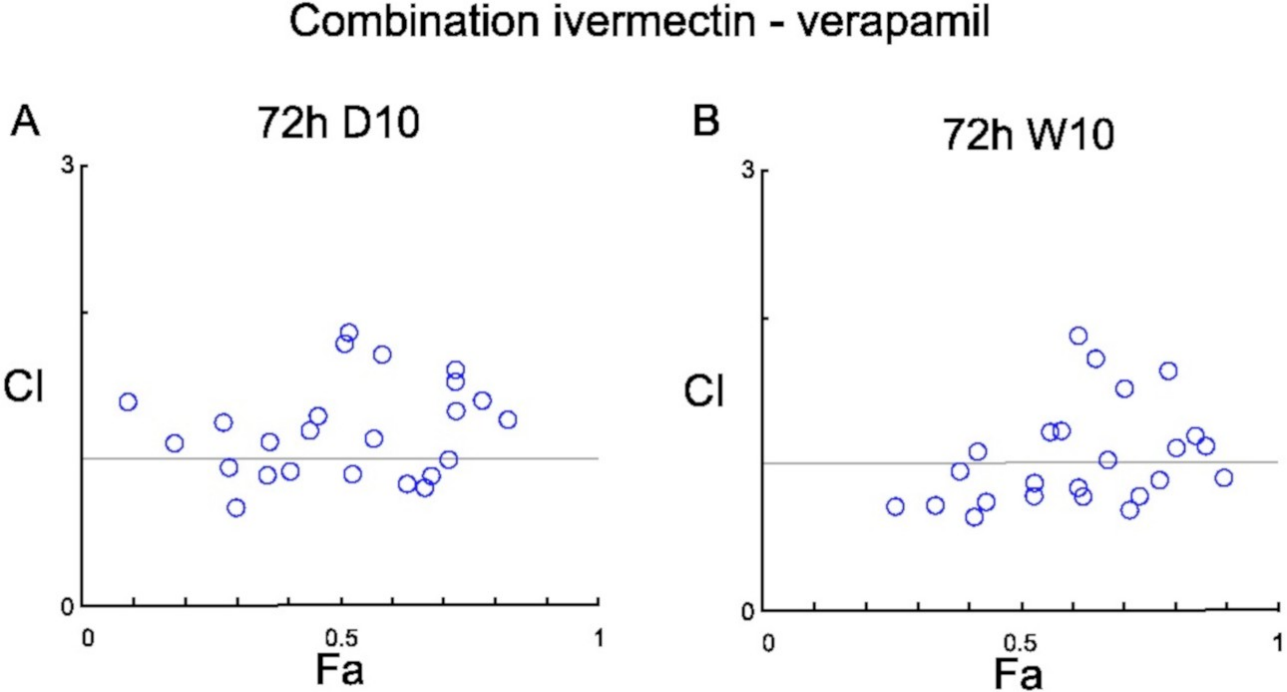
Combination Index plots for the anti-*P*. *falciparum* activity of Ivermectin (Ivm) in combination with Verapamil (Vpl) with an incubation time of 72h on asynchronous cultures of D10 (A) or W2 (B) strains. The data are representative of three independent experiments in duplicate for each combination.

Next, the effects of the combinations of the compounds under study on *P*. *falciparum* viability were assessed, analysing the results with the Chou-Talalay combination index (CI) method [34, 35]. The results of these analyses are shown in graphical form in Figs 2 and 3; the data recorded after the assays and the CI values are reported in S1 Text; the rankings in terms of the different degrees of synergism or antagonism, effected based on the CI values, are reported in S2 Text (rankings in this table are according to the CompuSyn and CalcuSyn manual, with the + symbols indicating synergism, and the–antagonism [35]). In Figs 2 and 3, the horizontal line at y = 1 indicates additivity; CI values below this line indicate synergism, while those above indicate antagonism. As observed in Fig 2 (as well as in S2 Text), the combination verapamil plus doxycycline showed a synergistic effect on W2 strain, at both time points. Most of the other combinations led to antagonistic interactions (e.g. ivermectin and doxycycline; elacridar and doxycycline). In a few cases CI values scattered around the additivity line were observed (Figs 2 and 3), with a balance of + and–symbols in the CI ranking table (S2 Text); in other words, no clear trend was observed toward the antagonistic or the synergistic sides.

## Discussion

In the assays using single drugs, no significant differences were observed between the CQ-resistant (W2) and CQ-sensitive (D10) strains of *P*. *falciparum*, when incubated for 72h with the presence of ivermectin, doxycycline or elacridar, meaning that the phenotype associated with chloroquine resistance has probably no effect on the efficacy of these drugs, at least in the conditions of the assays (Fig 1A–1C). On the contrary, W2 strain was significantly more susceptible to verapamil respect to D10 strain (Fig 1D). These results are comparable to those obtained by Martin et al. [20] with also very similar values of IC_50_ for verapamil alone on W2 and D6 strains (D6 is a CQ sensitive strain). It is also clear that the efficacy of verapamil against the parasite is 10-fold lower than that of ivermectin and elacridar at 72h incubation for the sensitive strain D10.

The results obtained for doxycycline were comparable to the ones obtained by Dahl et al. [15]. The almost 10-fold decrease in IC_50_ values from 72h to 96h incubation for both strains (Fig 1A) confirms that the principal effect of the antibiotic against the parasite is delayed, independently from the tested strain.

For ivermectin, the results obtained in this study show IC_50_ values that are different from those recorded in the few studies so far published. A study tested ivermectin *in vitro* against K1 strain of *P*. *falciparum* and found an IC_50_ value of ~9 μM [37], while another study tested it on different strains of *P*. *falciparum* (3D7 and JH26, CQ sensitive; Dd2, K1, JH1, and JH13, CQ resistant) and obtained IC_50_ values ranging from 20 to 365 nM [38]. No data are available in the literature for the *in vitro* ivermectin IC_50_ values on the strains tested in this study, nor for the delayed effect after 96h. As suggested by Pessanha de Carvalho et al. [38], the differences in IC_50_ values recorded in different studies might be due to different methodologies, different parasitaemia conditions, and obviously, as in this case, to the use of different strains.

IC_50_ values for verapamil were comparable to the ones in the literatures for 72h incubation [20]. No data are available for an incubation of 96h. Since this was the first time that elacridar was tested on *P*. *falciparum*, no data on this parasite are available for this drug in the literature, for a comparison with our results. Interestingly, the D10 strain of the parasite resulted more susceptible to both ivermectin and elacridar than the W2 strain, but only after 96h, opposite to the effect of verapamil.

As for drug combinations and CI analysis, doxycycline in combination with ivermectin acted in an antagonistic way for both strains. The antagonistic effect was more pronounced with 72h of incubation (Fig 2A–2B). These results may indicate that ivermectin does not target the transporters involved in the detoxification of doxycycline in the parasite.

The second efflux pump inhibitor tested in combination with doxycycline was verapamil. Current evidences suggest that the main target of this inhibitor in the parasite is the efflux pump of the digestive vacuole of *P*. *falciparum* (PfCRT). It is commonly held that only in resistant strains (such as W2), PfCRT effectively transports chloroquine out of the food vacuole; inhibition of this transporter, e.g. through verapamil, could thus be expected to restore drug sensitivity in resistant strains [20, 39]. Should PfCRT, either mutated for CQ resistance or not, be involved in the resistance to doxycycline, the verapamil assays on the two strains would end with comparable results (*i*.*e*. increased sensitivity to doxycycline when combined with verapamil). However, the results of the assays, at 72h, were rather different in the two strains: in D10 the effect of the combination was antagonistic, while in W2 was synergistic. The results at 96h showed variable results for D10, with synergism at low dosage of doxycycline, and antagonism at high dosages. In W2, the effect was strongly synergistic for most dosages. Besides the possible role of PfCRT, overall these results suggest that detoxification of doxycycline involves, at least in W2 strain, a mechanism that is inhibited by verapamil.

Based on these results, which indicate that the inhibition of drug efflux pumps might increase the efficacy of antimalarial drugs like doxycycline, elacridar, a third-generation ABC transporter inhibitor, was tested. The results from the elacridar plus doxycycline combination, with an incubation of 72h, showed an antagonistic effect for the D10 strains, and an additive one for W2. With an incubation time of 96h, the effect was antagonistic for both strains, with a less marked antagonism for D10. The results obtained thus suggest that elacridar and verapamil have different targets in *P*. *falciparum*.

The combination of verapamil plus ivermectin was also tested. Indeed, a potentiation of the effects of ivermectin by verapamil has been reported in several studies on metazoan parasites, such as nematodes, like *Caenorhabditis elegans* [40], *Haemonchus contortus* [41], and *Haemonchus placei* [42], as well as mosquitoes, like *Culex pipiens* [43]. Taken together, these evidences suggest that the action of verapamil, as an inhibitor of drug-detoxification, is conserved among distantly related organisms. However, our assays did not reveal any particular trend in the interaction between the two drugs, with the CI values scattered along the additivity line (Fig 3), with some indication for synergism only for low concentrations of verapamil in the W2 strain (S2 Text). The synergism verapamil+ivermectin could be explained by different hypothesis: i) verapamil may target a transporter that is not drug-specific, but that is responsible for the detoxification of many different drugs with no similarity in structure and site of action; ii) verapamil may target multiple transporters each specific for a different class of drugs; iii) verapamil may act on transporters or pumps that indirectly alter the susceptibility of *Plasmodium* to drugs (as proposed by Martiney et al. [44]).

On the other side, in the 72h assays, the medium was not changed. In addition, the 72h assays were done on asynchronous forms, while the 96h ones were on synchronized parasites. two time points are difficult to be compared, but the observations are anyway worth to be commented. The effect on the parasite appears to be immediate, in that the effects recorded at 96h (*i*.*e*. after 24h of drug exposure) were comparable to that recorded at 72h (*i*.*e*. after 72h of drug exposure). In other words, the further two days of incubation, up to the 72h time point, did not apparently lead to a difference in the efficacy of the drugs on the parasite (*i*.*e*. the IC_50_ values of the drug alone do not decrease between the two incubation times). The early effect of the drugs was also observed by Dahl et al. [15] with parasites incubated with doxycycline alone.

## Conclusions

Malaria impact on human health and quality of life has been reduced greatly during the last century, but its complete eradication is still far from being achieved. Inadequate funding [1, 45], climate changes [46, 47], resistance of malaria vectors towards the most commonly used classes of insecticides [4, 5], and the continuous emergence of parasite resistance towards antimalarial drugs [6, 48], are all factors that might be responsible for the recent increase in malaria incidence. These conditions, plus the ever-growing difficulty in the development of new drugs with an antimalarial effect, point to the need to develop novel strategies to fight this severe disease.

In this study, compounds that are already used in pharmacological therapy, in some cases already known for their antimalarial potential, have been tested, alone or in combination, with the purpose to contribute to the uncovering of novel potential combined treatments, as well as of possible negative drug interactions. The study provided novel results, on the tested drug combinations, with evidence for drug partners that act synergistically against the parasite (verapamil plus doxycycline in the CQ resistant strain), and partners that act in an antagonistic way, such as ivermectin plus doxycycline. This last result is relevant to the issue of mass ivermectin administration for onchocerciasis control, in regions where also malaria is endemic [30]. Considering the possibility that ivermectin and doxycycline are co-administered to the same patients, the antagonistic effects here recorded are worth of further investigations. On the other hand, since ivermectin has been shown to interfere with the normal life cycle of *P*. *falciparum*, inhibiting sporogony in the mosquito, influencing schizogony in the liver, and damaging both asexual and sexual stages in the blood of the host [25, 38], the search for molecules acting synergistically with this drug is also worth of further efforts. As for the verapamil plus doxycycline combination, the toxicity of verapamil excludes the possibility of exploiting this molecule as a partner drug in malaria control. However, the synergistic effect of this drug combination is coherent with the known mechanism of action of verapamil, as an inhibitor of ABC transporter efflux pumps. Therefore, more specific, or less toxic, efflux pump inhibitors could be investigated, and a better understanding of the mechanism of detoxification towards doxycycline could allow future development of novel partner drugs for this antibiotic. Unfortunately, the third-generation ABC transporter inhibitor here tested, elacridar, did not display any synergistic effect with doxycycline, rather the effect was antagonistic. These discordant results highlight the complexity of the system: the interplay between drug molecules and their putative targets, such as the transporters on organelles and on the different host and parasite membranes, is complex and poorly understood [49, 50]. Therefore, the results here presented, in term of synergism, additivity or antagonism, highlight issues that are worth of further investigations, but the translation to clinical application is far, and will obviously require *in vivo* studies.

## Supporting information

S1 Table IC_50_ values of the drugs against the two parasite strains at 72h and 96h. Data are expressed as IC_50_ ± SE of each drug alone on *P*. *falciparum* chloroquine-sensitive (D10) or chloroquine-resistant (W2) strains after 72h or 96h bioassays using the pLDH method. The results are the mean of at least three independent experiments in duplicate. Chloroquine used as control showed IC_50_ values of ~19 nM against D10 or ~440 nM against W2. DOX = doxycycline; IVM = Ivermectin; VPL = Verapamil; ELC = Elacridar.
(DOCX)

S1 Text Data used to build Figs 2 and 3 (Fa-CI plot) plus the concentration of drugs in the combined treatments. Combination index (CI) values were calculated using the equation: CI = C_A,x_/IC_x,A_ + C_B,x_/IC_x,B_ where IC_x,A_ and C_x,B_ are the concentrations of drug A and drug B used as a single agent to produce a given effect x and C_A,x_ and C_B,x_ are the concentrations of drug A and drug B in the combination to produce that same effect. Function of effect level (f_a_) represent the mortality induced by the combination of drugs at the selected combination (where 1 indicates 100% mortality. The values have been obtained from three independent replicas. Ivermectin (IVM); doxycycline (DOX); Elacridar (ELC); Verapamil (VPL). Drugs concentration are reported as μM.
(DOCX)

S2 Text Analyses for the anti-*P*. *falciparum* activity of drug combinations with an incubation time of 72h on asynchronous cultures of D10 or W2 strains, or 96h on synchronized cultures of D10 or W2 strains. Synergism, additivity or antagonism were classified on the basis of the Combination Index (CI) values and represented using the recommended symbols in Chou et al. (2005): CI value <0.1 very strong synergism (+++++), 0.1–0.3 strong synergism (++++), 0.3–0.7 synergism (+++), 0.7–0.85 moderate synergism (++), 0.85–0.90 slight synergism (+), 0.90–1.10 nearly additive (±), 1.1–1.20 slight antagonism (-), 1.20–1.45 moderate antagonism (- -), 1.45–3.3 antagonism (- - -), and >3.3 strong antagonism (- - - -), very strong antagonism (- - - - -) (Source: Compusyn and Calcusyn manual). Ivermectin (IVM); doxycycline (DOX); Elacridar (ELC); Verapamil (VPL).
(DOCX)
