# Supplementary material for: Effects of combined drug treatments on Plasmodium falciparum: In vitro assays with doxycycline, ivermectin and efflux pump inhibitors
Source: PLoS One. 2020 Apr 23;15(4):e0232171. doi: 10.1371/journal.pone.0232171 (PMC7179878; doi:10.1371/journal.pone.0232171)
Supplement: S1 Table — Data are expressed as IC50 ± SE of each drug alone on P. falciparum chloroquine-sensitive (D10) or chloroquine-resistant (W2) strains after 72h or 96h bioassays using the pLDH method. The results are the mean of at least three independent experiments in duplicate. Chloroquine used as control showed IC50 values of ~19 nM against D10 or ~440 nM against W2. DOX = doxycycline; IVM = Ivermectin; VPL = Verapamil; ELC = Elacridar. (DOCX) [file pone.0232171.s001.docx]

**S1 Table.** IC_50_ values of the drugs against the two parasite strains at 72h and 96h

|  | **DOX**  **IC_50_ (µM)** | | **IVM**  **IC_50_ (µM)** | | **VPL**  **IC_50_ (µM)** | | **ELC**  **IC_50_ (µM)** | |
| --- | --- | --- | --- | --- | --- | --- | --- | --- |
| ***P. falciparum***  **strain** | 72h | 96h | 72h | 96h | 72h | 96h | 72h | 96h |
| **D10** | 13,51 ± 0,36 | 1,90 ± 0,16 | 1,64 ± 0,23 | 1,31 ± 0,08 | 15,42 ± 1,82 | 15,09 ± 4,58 | 1,10 ± 0,11 | 0,89 ± 0,06 |
| **W2** | 12,77 ± 0,58 | 1,63 ± 0,15 | 1,87 ± 0,23 | 1,99 ± 0,25 | 6,56 ± 1,05 | 4,09 ± 0,98 | 1,17 ± 0,11 | 1,37 ± 0,08 |

Data are expressed as IC_50_ ± SE of each drug alone on *P. falciparum* chloroquine-sensitive (D10) or chloroquine-resistant (W2) strains after 72h or 96h bioassays using the pLDH method. The results are the mean of at least three independent experiments in duplicate. Chloroquine used as control showed IC_50_ values of ~19 nM against D10 or ~440 nM against W2. DOX=doxycycline; IVM=Ivermectin; VPL=Verapamil; ELC=Elacridar.
