# Supplementary material for: Effects of combined drug treatments on Plasmodium falciparum: In vitro assays with doxycycline, ivermectin and efflux pump inhibitors
Source: PLoS One. 2020 Apr 23;15(4):e0232171. doi: 10.1371/journal.pone.0232171 (PMC7179878; doi:10.1371/journal.pone.0232171)
Supplement: S1 Text — Combination index (CI) values were calculated using the equation: CI = CA,x/ICx,A + CB,x/ICx,B where ICx,A and Cx,B are the concentrations of drug A and drug B used as a single agent to produce a given effect x and CA,x and CB,x are the concentrations of drug A and drug B in the combination to produce that same effect. Function of effect level (fa) represent the mortality induced by the combination of drugs at the selected combination (where 1 indicates 100% mortality. The values have been obtained from three independent replicas. Ivermectin (IVM); doxycycline (DOX); Elacridar (ELC); Verapamil (VPL). Drugs concentration are reported as μM. (DOCX) [file pone.0232171.s002.docx]

**S1 Text.** Data used to build figure 2 and 3 (Fa-CI plot) plus the concentration of drugs in the combined treatments.

Combination index (CI) values were calculated using the equation:

CI =C_A,x_/IC_x,A_ + C_B,x_/IC_x,B_

where IC_x,A_ and C_x,B_ are the concentrations of drug A and drug B used as a single agent to produce a given effect x and C_A,x_ and C_B,x_ are the concentrations of drug A and drug B in the combination to produce that same effect.

Function of effect level (f_a_) represent the mortality induced by the combination of drugs at the selected combination (where 1 indicates 100% mortality.

The values have been obtained from three independent replicas.

Ivermectin (IVM); doxycycline (DOX); Elacridar (ELC); Verapamil (VPL). Drugs concentration are reported as µM.

**Table A: IVM+DOX 72h D10**

| **Dose DOX Dose IVM Effect (f_a_) CI** |
| --- |
| **(µM)** **(µM)** |
|  |
| 0,70 0,25 0,16 0,92 |
| 1,41 0,25 0,22 1,01 |
| 2,81 0,25 0,27 1,33 |
| 5,63 0,25 0,27 2,30 |
| 11,25 0,25 0,45 2,27 |
| 22,50 0,25 0,84 1,01 |
|  |
| 0,70 0,50 0,23 1,05 |
| 1,41 0,50 0,25 1,23 |
| 2,81 0,50 0,22 2,01 |
| 5,63 0,50 0,36 1,89 |
| 11,25 0,50 0,51 2,07 |
| 22,50 0,50 0,82 1,15 |
|  |
| 0,70 1,00 0,35 1,21 |
| 1,41 1,00 0,34 1,45 |
| 2,81 1,00 0,39 1,54 |
| 5,63 1,00 0,45 1,83 |
| 11,25 1,00 0,59 1,87 |
| 22,50 1,00 0,84 1,17 |
|  |
| 0,70 2,00 0,71 0,84 |
| 1,41 2,00 0,67 1,01 |
| 2,81 2,00 0,69 1,08 |
| 5,63 2,00 0,70 1,26 |
| 11,25 2,00 0,75 1,40 |
| 22,50 2,00 0,85 1,35 |

**Table B: IVM+DOX 72h W2**

| **Dose DOX Dose IVM Effect (f_a_) CI** |
| --- |
| **(µM)** **(µM)** |
|  |
| 0,70 0,25 0,10 0,82 |
| 1,41 0,25 0,15 0,99 |
| 2,81 0,25 0,15 1,67 |
| 5,63 0,25 0,22 2,23 |
| 11,25 0,25 0,39 2,34 |
|  |
| 0,70 0,50 0,16 0,92 |
| 1,41 0,50 0,17 1,23 |
| 2,81 0,50 0,27 1,29 |
| 5,63 0,50 0,26 2,20 |
| 11,25 0,50 0,39 2,52 |
|  |
| 0,70 1,00 0,20 1,40 |
| 1,41 1,00 0,23 1,53 |
| 2,81 1,00 0,30 1,70 |
| 5,63 1,00 0,29 2,45 |
| 11,25 1,00 0,46 2,50 |
|  |
| 0,70 2,00 0,33 2,04 |
| 1,41 2,00 0,40 1,98 |
| 2,81 2,00 0,43 2,16 |
| 5,63 2,00 0,47 2,45 |
| 11,25 2,00 0,54 2,82 |

**Table C: IVM+DOX 96h D10**

| **Dose DOX Dose IVM Effect (f_a_) CI** |
| --- |
| **(µM)** **(µM)** |
|  |
| 0,35 0,13 0,07 1,61 |
| 0,70 0,13 0,24 0,97 |
| 1,41 0,13 0,43 0,93 |
| 2,81 0,13 0,70 0,76 |
| 5,63 0,13 0,74 1,28 |
| 11,25 0,13 0,84 1,58 |
| 22,50 0,13 0,97 0,78 |
|  |
| 0,35 0,25 0,14 1,28 |
| 0,70 0,25 0,27 1,09 |
| 1,41 0,25 0,56 0,76 |
| 2,81 0,25 0,72 0,80 |
| 5,63 0,25 0,76 1,25 |
| 11,25 0,25 0,81 1,86 |
| 22,50 0,25 0,93 1,64 |
|  |
| 0,35 0,50 0,22 1,35 |
| 0,70 0,50 0,34 1,21 |
| 1,41 0,50 0,56 0,97 |
| 2,81 0,50 0,72 0,94 |
| 5,63 0,50 0,77 1,30 |
| 11,25 0,50 0,85 1,68 |
| 22,50 0,50 0,95 1,37 |
|  |
| 0,35 1,00 0,41 1,42 |
| 0,70 1,00 0,45 1,47 |
| 1,41 1,00 0,58 1,33 |
| 2,81 1,00 0,76 1,10 |
| 5,63 1,00 0,85 1,14 |
| 11,25 1,00 0,89 1,42 |
| 22,50 1,00 0,96 1,27 |

**Table D: IVM+DOX 96h W2**

| **Dose DOX Dose IVM Effect (f_a_) CI** |
| --- |
| **(µM)** **(µM)** |
|  |
| 0,35 0,13 0,05 1,55 |
| 0,70 0,13 0,27 0,74 |
| 1,41 0,13 0,39 0,90 |
| 2,81 0,13 0,67 0,75 |
| 5,63 0,13 0,76 1,04 |
| 11,25 0,13 0,83 1,44 |
|  |
| 0,35 0,25 0,11 1,13 |
| 0,70 0,25 0,25 1,00 |
| 1,41 0,25 0,51 0,80 |
| 2,81 0,25 0,65 0,92 |
| 5,63 0,25 0,74 1,19 |
| 11,25 0,25 0,78 1,93 |
|  |
| 0,35 0,50 0,16 1,41 |
| 0,70 0,50 0,27 1,33 |
| 1,41 0,50 0,52 1,06 |
| 2,81 0,50 0,66 1,11 |
| 5,63 0,50 0,73 1,44 |
| 11,25 0,50 0,82 1,78 |
|  |
| 0,35 1,00 0,28 1,80 |
| 0,70 1,00 0,35 1,79 |
| 1,41 1,00 0,55 1,52 |
| 2,81 1,00 0,71 1,40 |
| 5,63 1,00 0,77 1,67 |
| 11,25 1,00 0,87 1,71 |

**Table E: VPL+DOX 72h D10**

| **Dose DOX Dose VPL Effect (f_a_) CI** |
| --- |
| **(µM)** **(µM)** |
|  |
| 0,70 0,62 0,12 0,71 |
| 1,41 0,62 0,11 1,20 |
| 2,81 0,62 0,16 1,56 |
| 5,63 0,62 0,23 2,01 |
| 11,25 0,62 0,44 1,88 |
|  |
| 0,70 1,25 0,13 0,96 |
| 1,41 1,25 0,11 1,56 |
| 2,81 1,25 0,18 1,55 |
| 5,63 1,25 0,30 1,65 |
| 11,25 1,25 0,54 1,45 |
|  |
| 0,70 2,50 0,26 0,77 |
| 1,41 2,50 0,29 0,86 |
| 2,81 2,50 0,34 1,04 |
| 5,63 2,50 0,43 1,23 |
| 11,25 2,50 0,67 1,02 |
|  |
| 0,70 5,00 0,33 1,01 |
| 1,41 5,00 0,38 0,98 |
| 2,81 5,00 0,41 1,14 |
| 5,63 5,00 0,54 1,11 |
| 11,25 5,00 0,80 0,70 |
|  |
| 0,70 10,00 0,40 1,45 |
| 1,41 10,00 0,46 1,31 |
| 2,81 10,00 0,49 1,41 |
| 5,63 10,00 0,56 1,42 |
| 11,25 10,00 0,81 0,84 |

**Table F: VPL+DOX 72h W2**

| **Dose DOX Dose VPL Effect (f_a_) CI** |
| --- |
| **(µM)** **(µM)** |
|  |
| 0,70 0,63 0,18 0,44 |
| 1,41 0,63 0,17 0,67 |
| 2,81 0,63 0,23 0,87 |
| 5,63 0,63 0,35 1,10 |
| 11,25 0,63 0,57 1,25 |
| 22,50 0,63 0,95 0,54 |
|  |
| 0,70 1,25 0,22 0,56 |
| 1,41 1,25 0,27 0,61 |
| 2,81 1,25 0,35 0,73 |
| 5,63 1,25 0,46 0,94 |
| 11,25 1,25 0,68 0,99 |
| 22,50 1,25 0,98 0,36 |
|  |
| 0,70 2,50 0,41 0,48 |
| 1,41 2,50 0,44 0,55 |
| 2,81 2,50 0,49 0,66 |
| 5,63 2,50 0,57 0,83 |
| 11,25 2,50 0,73 0,92 |
| 22,50 2,50 0,99 0,24 |
|  |
| 0,70 5,00 0,54 0,58 |
| 1,41 5,00 0,57 0,62 |
| 2,81 5,00 0,60 0,71 |
| 5,63 5,00 0,65 0,84 |
| 11,25 5,00 0,86 0,64 |
| 22,50 5,00 0,98 0,33 |
|  |
| 0,70 10,00 0,58 0,98 |
| 1,41 10,00 0,62 0,92 |
| 2,81 10,00 0,69 0,85 |
| 11,25 10,00 0,89 0,66 |
| 22,50 10,00 0,99 0,23 |

**Table G: VPL+DOX 96h D10**

| **Dose DOX Dose VPL Effect (f_a_) CI** |
| --- |
| **(µM)** **(µM)** |
|  |
| 0,35 0,31 0,18 0,37 |
| 0,70 0,31 0,29 0,42 |
| 1,41 0,31 0,52 0,34 |
| 2,81 0,31 0,58 0,89 |
| 5,63 0,31 0,62 2,71 |
| 11,25 0,31 0,72 5,22 |
|  |
| 0,35 0,63 0,17 0,53 |
| 0,70 0,63 0,28 0,52 |
| 1,41 0,63 0,55 0,30 |
| 2,81 0,63 0,62 0,71 |
| 5,63 0,63 0,68 1,86 |
| 11,25 0,63 0,78 3,14 |
|  |
| 0,35 1,25 0,19 0,62 |
| 0,70 1,25 0,33 0,44 |
| 1,41 1,25 0,55 0,32 |
| 2,81 1,25 0,63 0,66 |
| 5,63 1,25 0,70 1,56 |
| 11,25 1,25 0,80 2,76 |
|  |
| 0,35 2,50 0,34 0,35 |
| 0,70 2,50 0,41 0,37 |
| 1,41 2,50 0,48 0,55 |
| 2,81 2,50 0,57 1,04 |
| 5,63 2,50 0,67 1,98 |
| 11,25 2,50 0,82 2,21 |
|  |
| 0,35 5,00 0,43 0,39 |
| 0,70 5,00 0,47 0,40 |
| 1,41 5,00 0,44 0,87 |
| 2,81 5,00 0,53 1,40 |
| 5,63 5,00 0,56 4,24 |
| 11,25 5,00 0,84 1,81 |

**Table H: VPL+DOX 96h W2**

| **Dose DOX Dose VPL Effect (f_a_) CI** |
| --- |
| **(µM)** **(µM)** |
|  |
| 0,35 0,31 0,19 0,39 |
| 0,70 0,31 0,28 0,40 |
| 1,41 0,31 0,46 0,32 |
| 2,81 0,31 0,63 0,32 |
| 5,63 0,31 0,78 0,30 |
| 11,25 0,31 0,84 0,55 |
|  |
| 0,35 0,63 0,36 0,23 |
| 0,70 0,63 0,32 0,40 |
| 1,41 0,63 0,47 0,36 |
| 2,81 0,63 0,71 0,20 |
| 5,63 0,63 0,81 0,25 |
| 11,25 0,63 0,85 0,53 |
|  |
| 0,35 1,25 0,37 0,41 |
| 0,70 1,25 0,45 0,35 |
| 1,41 1,25 0,62 0,23 |
| 2,81 1,25 0,74 0,21 |
| 5,63 1,25 0,83 0,22 |
| 11,25 1,25 0,89 0,32 |
|  |
| 0,35 2,50 0,51 0,47 |
| 0,70 2,50 0,57 0,41 |
| 1,41 2,50 0,67 0,32 |
| 2,81 2,50 0,75 0,29 |
| 5,63 2,50 0,85 0,24 |
| 11,25 2,50 0,94 0,13 |
|  |
| 0,35 5,00 0,76 0,36 |
| 0,70 5,00 0,71 0,45 |
| 1,41 5,00 0,78 0,34 |
| 2,81 5,00 0,82 0,30 |
| 5,63 5,00 0,87 0,27 |
| 11,25 5,00 0,96 0,10 |

**Table I: ELC+DOX 72h D10**

| **Dose DOX Dose ELC Effect (f_a_) CI** |
| --- |
| **(µM)** **(µM)** |
|  |
| 0,35 0,22 0,03 1,31 |
| 0,70 0,22 0,07 1,20 |
| 1,41 0,22 0,14 1,27 |
| 2,81 0,22 0,16 1,91 |
| 5,63 0,22 0,22 2,94 |
| 11,25 0,22 0,53 3,69 |
|  |
| 0,35 0,44 0,01 3,79 |
| 0,70 0,44 0,16 1,36 |
| 1,41 0,44 0,24 1,40 |
| 2,81 0,44 0,24 2,03 |
| 5,63 0,44 0,29 2,97 |
| 11,25 0,44 0,58 3,69 |
|  |
| 0,35 0,89 0,47 1,12 |
| 0,70 0,89 0,51 1,16 |
| 1,41 0,89 0,57 1,26 |
| 2,81 0,89 0,58 1,65 |
| 5,63 0,89 0,63 2,32 |
| 11,25 0,89 0,78 3,06 |
|  |
| 0,35 1,77 0,80 1,14 |
| 0,70 1,77 0,76 1,33 |
| 1,41 1,77 0,79 1,38 |
| 2,81 1,77 0,79 1,69 |
| 5,63 1,77 0,83 2,10 |
| 11,25 1,77 0,84 3,17 |

**Table J: ELC+DOX 72h W2**

| **Dose DOX Dose ELC Effect (f_a_) CI** |
| --- |
| **(µM)** **(µM)** |
|  |
| 0,35 0,11 0,06 1,06 |
| 0,70 0,11 0,19 0,66 |
| 1,41 0,11 0,30 0,68 |
| 2,81 0,11 0,35 0,99 |
| 5,63 0,11 0,46 1,25 |
| 11,25 0,11 0,71 1,03 |
|  |
| 0,35 0,22 0,11 1,06 |
| 0,70 0,22 0,23 0,79 |
| 1,41 0,22 0,29 0,88 |
| 2,81 0,22 0,38 1,05 |
| 5,63 0,22 0,48 1,29 |
| 11,25 0,22 0,71 1,13 |
|  |
| 0,35 0,44 0,21 1,13 |
| 0,70 0,44 0,29 1,02 |
| 1,41 0,44 0,35 1,05 |
| 2,81 0,44 0,43 1,16 |
| 5,63 0,44 0,50 1,45 |
| 11,25 0,44 0,73 1,15 |
|  |
| 0,35 0,89 0,71 0,59 |
| 0,70 0,89 0,55 0,93 |
| 1,41 0,89 0,58 0,96 |
| 2,81 0,89 0,66 0,94 |
| 5,63 0,89 0,66 1,24 |
| 11,25 0,89 0,80 1,09 |
|  |
| 0,35 1,77 0,66 1,30 |
| 0,70 1,77 0,67 1,33 |
| 1,41 1,77 0,69 1,31 |
| 2,81 1,77 0,74 1,24 |
| 5,63 1,77 0,76 1,39 |
| 11,25 1,77 0,80 1,50 |

**Table K: ELC+DOX 96h D10**

| **Dose DOX Dose ELC Effect (f_a_) CI** |
| --- |
| **(µM)** **(µM)** |
|  |
| 0,70 0,06 0,15 1,11 |
| 1,41 0,06 0,45 0,78 |
| 2,81 0,06 0,69 0,78 |
| 5,63 0,06 0,76 1,22 |
| 11,25 0,06 0,80 2,01 |
|  |
| 0,70 0,11 0,24 1,02 |
| 1,41 0,11 0,57 0,70 |
| 2,81 0,11 0,73 0,78 |
| 5,63 0,11 0,80 1,10 |
| 11,25 0,11 0,84 1,75 |
|  |
| 0,70 0,22 0,24 1,43 |
| 1,41 0,22 0,57 0,92 |
| 2,81 0,22 0,71 0,98 |
| 5,63 0,22 0,79 1,30 |
| 11,25 0,22 0,82 2,05 |
|  |
| 0,70 0,44 0,43 1,51 |
| 1,41 0,44 0,66 1,13 |
| 2,81 0,44 0,77 1,11 |
| 5,63 0,44 0,81 1,48 |
| 11,25 0,44 0,84 2,09 |

**Table L: ELC+DOX 96h W2**

| **Dose DOX Dose ELC Effect (f_a_) CI** |
| --- |
| **(µM)** **(µM)** |
|  |
| 0,70 0,06 0,06 2,29 |
| 1,41 0,06 0,16 2,15 |
| 2,81 0,06 0,42 1,93 |
| 5,63 0,06 0,76 1,70 |
| 11,25 0,06 0,87 2,27 |
| 22,50 0,06 0,93 3,12 |
|  |
| 0,70 0,11 0,10 2,22 |
| 1,41 0,11 0,25 1,81 |
| 2,81 0,11 0,52 1,64 |
| 5,63 0,11 0,79 1,58 |
| 11,25 0,11 0,88 2,19 |
| 22,50 0,11 0,94 2,91 |
|  |
| 0,70 0,22 0,10 3,06 |
| 1,41 0,22 0,27 2,10 |
| 2,81 0,22 0,55 1,72 |
| 5,63 0,22 0,78 1,71 |
| 11,25 0,22 0,88 2,21 |
| 22,50 0,22 0,94 2,94 |
|  |
| 0,70 0,44 0,37 1,70 |
| 1,41 0,44 0,46 1,74 |
| 2,81 0,44 0,65 1,63 |
| 5,63 0,44 0,80 1,76 |
| 11,25 0,44 0,88 2,31 |
| 22,50 0,44 0,94 2,97 |

**Table M: IVM+VPL 72h D10**

| **Dose IVM Dose VPL Effect (f_a_) CI** |
| --- |
| **(µM)** **(µM)** |
|  |
| 0,16 0,94 0,09 1,38 |
| 0,16 1,88 0,30 0,66 |
| 0,16 3,75 0,36 0,88 |
| 0,16 7,50 0,44 1,19 |
| 0,16 15,00 0,51 1,78 |
| 0,16 30,00 0,73 1,54 |
|  |
| 0,31 0,94 0,18 1,10 |
| 0,31 1,88 0,29 0,94 |
| 0,31 3,75 0,41 0,92 |
| 0,31 7,50 0,46 1,29 |
| 0,31 15,00 0,52 1,85 |
| 0,31 30,00 0,73 1,61 |
|  |
| 0,63 0,94 0,28 1,24 |
| 0,63 1,88 0,36 1,11 |
| 0,63 3,75 0,52 0,90 |
| 0,63 7,50 0,56 1,14 |
| 0,63 15,00 0,58 1,72 |
| 0,63 30,00 0,78 1,39 |
|  |
| 1,25 0,94 0,63 0,83 |
| 1,25 1,88 0,67 0,81 |
| 1,25 3,75 0,68 0,89 |
| 1,25 7,50 0,71 1,00 |
| 1,25 15,00 0,72 1,33 |
| 1,25 30,00 0,83 1,27 |

**Table N: IVM+VPL 72h W2**

| **Dose IVM Dose VPL Effect (f_a_) CI** |
| --- |
| **(µM)** **(µM)** |
|  |
| 0,16 0,94 0,27 0,70 |
| 0,16 1,88 0,42 0,63 |
| 0,16 3,75 0,43 1,08 |
| 0,16 7,50 0,57 1,22 |
| 0,16 15,00 0,63 1,87 |
| 0,16 30,00 0,81 1,66 |
|  |
| 0,31 0,94 0,34 0,72 |
| 0,31 1,88 0,44 0,74 |
| 0,31 3,75 0,54 0,86 |
| 0,31 7,50 0,59 1,23 |
| 0,31 15,00 0,66 1,73 |
| 0,31 30,00 0,86 1,22 |
|  |
| 0,63 0,94 0,39 0,96 |
| 0,63 1,88 0,54 0,78 |
| 0,63 3,75 0,64 0,79 |
| 0,63 7,50 0,68 1,03 |
| 0,63 15,00 0,72 1,53 |
| 0,63 30,00 0,88 1,14 |
|  |
| 1,25 0,94 0,63 0,84 |
| 1,25 1,88 0,73 0,69 |
| 1,25 3,75 0,75 0,78 |
| 1,25 7,50 0,79 0,89 |
| 1,25 15,00 0,82 1,12 |
| 1,25 30,00 0,91 0,92 |
