# Supplementary material for: Effects of combined drug treatments on Plasmodium falciparum: In vitro assays with doxycycline, ivermectin and efflux pump inhibitors
Source: PLoS One. 2020 Apr 23;15(4):e0232171. doi: 10.1371/journal.pone.0232171 (PMC7179878; doi:10.1371/journal.pone.0232171)
Supplement: S2 Text — Synergism, additivity or antagonism were classified on the basis of the Combination Index (CI) values and represented using the recommended symbols in Chou et al. (2005): CI value <0.1 very strong synergism (+++++), 0.1–0.3 strong synergism (++++), 0.3–0.7 synergism (+++), 0.7–0.85 moderate synergism (++), 0.85–0.90 slight synergism (+), 0.90–1.10 nearly additive (±), 1.1–1.20 slight antagonism (-), 1.20–1.45 moderate antagonism (- -), 1.45–3.3 antagonism (- - -), and >3.3 strong antagonism (- - - -), very strong antagonism (- - - - -) (Source: Compusyn and Calcusyn manual). Ivermectin (IVM); doxycycline (DOX); Elacridar (ELC); Verapamil (VPL). (DOCX) [file pone.0232171.s003.docx]

**S2 Text.**

Analyses for the anti-*P. falciparum* activity of drug combinations with an incubation time of 72h on asynchronous cultures of D10 or W2 strains, or 96h on synchronized cultures of D10 or W2 strains. Synergism, additivity or antagonism were classified on the basis of the Combination Index (CI) values and represented using the recommended symbols in Chou et al. (2005): CI value <0.1 very strong synergism (+++++), 0.1–0.3 strong synergism (++++), 0.3–0.7 synergism (+++), 0.7–0.85 moderate synergism (++), 0.85-0.90 slight synergism (+), 0.90-1.10 nearly additive (±), 1.1–1.20 slight antagonism (-), 1.20–1.45 moderate antagonism (--), 1.45–3.3 antagonism (---), and >3.3 strong antagonism (----), very strong antagonism (-----) (Source: Compusyn and Calcusyn manual).

Ivermectin (IVM); doxycycline (DOX); Elacridar (ELC); Verapamil (VPL).

**Table A: IVM+DOX 72h**

|  |  |  | CI values and type of interaction | | | | | | | | | |  |
| --- | --- | --- | --- | --- | --- | --- | --- | --- | --- | --- | --- | --- | --- |
| Strain  72h | Doxycycline  (µM) |  | at 0,25 µM IVM | | at 0,50 µM IVM | | at 1,00 µM IVM | | | at 2,00 µM IVM | | |  |
| D10 | 0,70 |  | 0,92 | ± | 1,05 | ± | | 1,21 | -- | | 0,84 | ++ | |
|  | 1,41 |  | 1,01 | ± | 1,22 | -- | | 1,44 | -- | | 1,01 | ± | |
|  | 2,81 |  | 1,33 | -- | 2,01 | --- | | 1,53 | --- | | 1,08 | ± | |
|  | 5,63 |  | 2,30 | --- | 1,89 | --- | | 1,82 | --- | | 1,26 | -- | |
|  | 11,25 |  | 2,27 | --- | 2,06 | --- | | 1,86 | --- | | 1,40 | -- | |
|  | 22,50 |  | 1,01 | ± | 1,14 | - | | 1,17 | - | | 1,34 | -- | |
|  |  |  |  |  |  |  | |  |  | |  |  | |
| W2 | 0,70 |  | 0,82 | ++ | 0,92 | ± | | 1,40 | -- | | 2,04 | --- | |
|  | 1,41 |  | 0,99 | ± | 1,23 | -- | | 1,53 | --- | | 1,98 | --- | |
|  | 2,81 |  | 1,67 | --- | 1,29 | -- | | 1,70 | --- | | 2,16 | --- | |
|  | 5,63 |  | 2,23 | --- | 2,20 | --- | | 2,45 | --- | | 2,45 | --- | |
|  | 11,25 |  | 2,34 | --- | 2,52 | --- | | 2,50 | --- | | 2,82 | --- | |
|  |  |  |  |  |  |  | |  |  | |  |  | |

**Table B: IVM+DOX 96h**

|  |  |  | CI values and type of interaction | | | | | | | | | |  |
| --- | --- | --- | --- | --- | --- | --- | --- | --- | --- | --- | --- | --- | --- |
| Strain  96h | Doxycycline  (µM) |  | at 0,12 µM IVM | | at 0,25 µM IVM | | at 0,50 µM IVM | | | at 1,00 µM IVM | | |  |
| D10 | 0,35 |  | 1,61 | --- | 1,28 | -- | | 1,35 | -- | | 1,42 | -- | |
|  | 0,70 |  | 0,97 | ± | 1,09 | ± | | 1,21 | -- | | 1,47 | --- | |
|  | 1,41 |  | 0,93 | ± | 0,76 | ++ | | 0,97 | ± | | 1,33 | -- | |
|  | 2,81 |  | 0,76 | ++ | 0,80 | ++ | | 0,94 | ± | | 1,10 | ± | |
|  | 5,63 |  | 1,28 | -- | 1,25 | -- | | 1,30 | -- | | 1,14 | - | |
|  | 11,25 |  | 1,58 | --- | 1,86 | --- | | 1,68 | --- | | 1,42 | -- | |
|  | 22,50 |  | 0,78 | ++ | 1,64 | --- | | 1,37 | -- | | 1,27 | -- | |
|  |  |  |  |  |  |  | |  |  | |  |  | |
| W2 | 0,35 |  | 1,55 | --- | 1,13 | - | | 1,41 | -- | | 1,80 | --- | |
|  | 0,70 |  | 0,74 | ++ | 1,00 | ± | | 1,33 | -- | | 1,79 | --- | |
|  | 1,41 |  | 0,90 | + | 0,80 | ++ | | 1,06 | ± | | 1,52 | --- | |
|  | 2,81 |  | 0,75 | ++ | 0,92 | ± | | 1,11 | - | | 1,40 | -- | |
|  | 5,63 |  | 1,04 | ± | 1,19 | - | | 1,44 | -- | | 1,67 | --- | |
|  | 11,25 |  | 1,44 | -- | 1,93 | --- | | 1,78 | --- | | 1,71 | --- | |
|  |  |  |  |  |  |  | |  |  | |  |  | |

**Table C: VPL+DOX 72h**

|  |  |  | CI values and type of interaction | | | | | | | | | | |  |  | |  |
| --- | --- | --- | --- | --- | --- | --- | --- | --- | --- | --- | --- | --- | --- | --- | --- | --- | --- |
| Strain  72h | Doxycycline  (µM) |  | at 0,62 µM VPL | | at 1,25 µM VPL | | | at 2,50 µM VPL | | | at 5,00 µM VPL | | | At 10,00 µM VPL | | |  |
| D10 | 0,70 |  | 0,71 | ++ | | 0,96 | ± | | 0,77 | ++ | | 1,01 | ± | 1,45 | | --- | |
|  | 1,41 |  | 1,20 | - | | 1,56 | --- | | 0,86 | + | | 0,98 | ± | 1,31 | | -- | |
|  | 2,81 |  | 1,56 | --- | | 1,55 | --- | | 1,04 | ± | | 1,14 | - | 1,41 | | -- | |
|  | 5,63 |  | 2,01 | --- | | 1,65 | --- | | 1,23 | -- | | 1,11 | - | 1,42 | | -- | |
|  | 11,25 |  | 1,88 | --- | | 1,45 | -- | | 1,02 | ± | | 0,70 | +++ | 0,84 | | ++ | |
|  |  |  |  |  | |  |  | |  |  | |  |  |  | |  | |
| W2 | 0,70 |  | 0,44 | +++ | | 0,56 | +++ | | 0,48 | +++ | | 0,58 | +++ | 0,98 | | ± | |
|  | 1,41 |  | 0,67 | +++ | | 0,61 | +++ | | 0,55 | +++ | | 0,62 | +++ | 0,92 | | ± | |
|  | 2,81 |  | 0,87 | + | | 0,73 | ++ | | 0,66 | +++ | | 0,71 | ++ | 0,85 | | ++ | |
|  | 5,63 |  | 1,10 | - | | 0,94 | ± | | 0,83 | ++ | | 0,84 | ++ |  | |  | |
|  | 11,25 |  | 1,25 | -- | | 0,99 | ± | | 0,92 | ± | | 0,64 | +++ | 0,66 | | +++ | |
|  | 22,50 |  | 0,54 | +++ | | 0,36 | +++ | | 0,24 | ++++ | | 0,33 | +++ | 0,23 | | ++++ | |
|  |  |  |  |  | |  |  | |  |  | |  |  |  | |  | |

**Table D: VPL+DOX 96h**

|  |  |  | CI values and type of interaction | | | | | | | | | | |  |  | |  |
| --- | --- | --- | --- | --- | --- | --- | --- | --- | --- | --- | --- | --- | --- | --- | --- | --- | --- |
| Strain  96h | Doxycycline  (µM) |  | At 0,31 µM VPL | | at 0,62 µM VPL | | | at 1,25 µM VPL | | | at 2,50 µM VPL | | | at 5,00 µM VPL | | |  |
| D10 | 0,35 |  | 0,37 | +++ | | 0,53 | +++ | | 0,62 | +++ | | 0,35 | +++ | 0,39 | | +++ | |
|  | 0,70 |  | 0,42 | +++ | | 0,52 | +++ | | 0,44 | +++ | | 0,37 | +++ | 0,40 | | +++ | |
|  | 1,41 |  | 0,34 | +++ | | 0,30 | +++ | | 0,32 | +++ | | 0,55 | +++ | 0,87 | | + | |
|  | 2,81 |  | 0,89 | + | | 0,71 | ++ | | 0,66 | +++ | | 1,04 | ± | 1,40 | | -- | |
|  | 5,63 |  | 2,71 | --- | | 1,86 | --- | | 1,56 | --- | | 1,98 | --- | 4,24 | | ---- | |
|  | 11,25 |  | 5,22 | ---- | | 3,14 | --- | | 2,76 | --- | | 2,21 | --- | 1,81 | | --- | |
|  |  |  |  |  | |  |  | |  |  | |  |  |  | |  | |
|  |  |  |  |  | |  |  | |  |  | |  |  |  | |  | |
| W2 | 0,35 |  | 0,39 | +++ | | 0,23 | ++++ | | 0,41 | +++ | | 0,47 | +++ | 0,36 | | +++ | |
|  | 0,70 |  | 0,40 | +++ | | 0,40 | +++ | | 0,35 | +++ | | 0,41 | +++ | 0,45 | | +++ | |
|  | 1,41 |  | 0,32 | +++ | | 0,36 | +++ | | 0,23 | ++++ | | 0,32 | +++ | 0,34 | | +++ | |
|  | 2,81 |  | 0,32 | +++ | | 0,20 | ++++ | | 0,21 | ++++ | | 0,29 | ++++ | 0,30 | | +++ | |
|  | 5,63 |  | 0,30 | +++ | | 0,25 | ++++ | | 0,22 | ++++ | | 0,24 | ++++ | 0,27 | | ++++ | |
|  | 11,25 |  | 0,55 | +++ | | 0,53 | +++ | | 0,32 | +++ | | 0,13 | ++++ | 0,10 | | ++++ | |
|  |  |  |  |  | |  |  | |  |  | |  |  |  | |  | |

**Table E: ELC+DOX 72h**

|  |  |  | CI values and type of interaction | | | | | | | | | | | | |  |
| --- | --- | --- | --- | --- | --- | --- | --- | --- | --- | --- | --- | --- | --- | --- | --- | --- |
| Strain  72h | Doxycycline  (µM) |  | At 0,11 µM ELC | | At 0,22 µM ELC | | At 0,44 µM ELC | | | At 0,89 µM ELC | | | At 1,77 µM ELC | | |  |
| D10 | 0,35 |  |  |  | 1,31 | -- | | 3,79 | ---- | | 1,12 | - | | 1,14 | - | |
|  | 0,70 |  |  |  | 1,20 | -- | | 1,36 | -- | | 1,16 | - | | 1,33 | -- | |
|  | 1,41 |  |  |  | 1,27 | -- | | 1,40 | -- | | 1,26 | -- | | 1,38 | -- | |
|  | 2,81 |  |  |  | 1,91 | --- | | 2,03 | --- | | 1,65 | --- | | 1,69 | --- | |
|  | 5,63 |  |  |  | 2,94 | --- | | 2,97 | --- | | 2,32 | --- | | 2,10 | --- | |
|  | 11,25 |  |  |  | 3,69 | ---- | | 3,69 | ---- | | 3,06 | --- | | 3,17 | --- | |
|  |  |  |  |  |  |  | |  |  | |  |  | |  |  | |
| W2 | 0,35 |  | 1,06 | ± | 1,06 | ± | | 1,13 | - | | 0,59 | +++ | | 1,30 | -- | |
|  | 0,70 |  | 0,66 | +++ | 0,79 | ++ | | 1,02 | ± | | 0,93 | ± | | 1,33 | -- | |
|  | 1,41 |  | 0,68 | +++ | 0,88 | + | | 1,05 | ± | | 0,96 | ± | | 1,31 | -- | |
|  | 2,81 |  | 0,99 | ± | 1,05 | ± | | 1,16 | - | | 0,94 | ± | | 1,24 | -- | |
|  | 5,63 |  | 1,25 | -- | 1,29 | -- | | 1,45 | --- | | 1,24 | -- | | 1,39 | -- | |
|  | 11,25 |  | 1,03 | ± | 1,13 | - | | 1,15 | - | | 1,09 | ± | | 1,50 | --- | |
|  |  |  |  |  |  |  | |  |  | |  |  | |  |  | |

**Table F: ELC+DOX 96h**

|  |  |  | CI values and type of interaction | | | | | | | | | |  |
| --- | --- | --- | --- | --- | --- | --- | --- | --- | --- | --- | --- | --- | --- |
| Strain  96h | Doxycycline  (µM) |  | At 0,06 µM ELC | | At 0,11 µM ELC | | At 0,22 µM ELC | | | At 0,44 µM ELC | | |  |
| D10 | 0,70 |  | 1,11 | - | 1,02 | ± | | 1,43 | -- | | 1,51 | --- | |
|  | 1,41 |  | 0,78 | ++ | 0,70 | +++ | | 0,92 | ± | | 1,13 | - | |
|  | 2,81 |  | 0,78 | ++ | 0,78 | ++ | | 0,98 | ± | | 1,11 | - | |
|  | 5,63 |  | 1,22 | -- | 1,10 | - | | 1,30 | -- | | 1,48 | --- | |
|  | 11,25 |  | 2,01 | --- | 1,75 | --- | | 2,05 | --- | | 2,09 | --- | |
|  |  |  |  |  |  |  | |  |  | |  |  | |
| W2 | 0,70 |  | 2,29 | --- | 2,22 | --- | | 3,06 | --- | | 1,70 | --- | |
|  | 1,41 |  | 2,15 | --- | 1,81 | --- | | 2,10 | --- | | 1,74 | --- | |
|  | 2,81 |  | 1,93 | --- | 1,64 | --- | | 1,72 | --- | | 1,63 | --- | |
|  | 5,63 |  | 1,70 | --- | 1,58 | --- | | 1,71 | --- | | 1,76 | --- | |
|  | 11,25 |  | 2,27 | --- | 2,19 | --- | | 2,21 | --- | | 2,31 | --- | |
|  | 22,50 |  | 3,12 | --- | 2,91 | --- | | 2,94 | --- | | 2,97 | --- | |
|  |  |  |  |  |  |  | |  |  | |  |  | |

**Table G: IVM+VPL 72h**

|  |  |  | CI values and type of interaction | | | | | | | | | |  |
| --- | --- | --- | --- | --- | --- | --- | --- | --- | --- | --- | --- | --- | --- |
| Strain  72h | Verapamil  (µM) |  | At 0,16 µM IVM | | At 0,31 µM IVM | | At 0,62 µM IVM | | | At 1,25 µM IVM | | |  |
| D10 | 0,94 |  | 1,38 | -- | 1,10 | - | | 1,24 | -- | | 0,83 | ++ | |
|  | 1,87 |  | 0,66 | +++ | 0,94 | ± | | 1,11 | - | | 0,81 | ++ | |
|  | 3,75 |  | 0,88 | + | 0,92 | ± | | 0,90 | + | | 0,89 | + | |
|  | 7,50 |  | 1,19 | - | 1,29 | -- | | 1,14 | - | | 1,00 | ± | |
|  | 15,00 |  | 1,78 | --- | 1,85 | --- | | 1,72 | --- | | 1,33 | -- | |
|  | 30,00 |  | 1,54 | --- | 1,61 | --- | | 1,39 | -- | | 1,27 | -- | |
|  |  |  |  |  |  |  | |  |  | |  |  | |
| W2 | 0,94 |  | 0,70 | +++ | 0,72 | ++ | | 0,96 | ± | | 0,84 | ++ | |
|  | 1,87 |  | 0,63 | +++ | 0,74 | ++ | | 0,78 | ++ | | 0,69 | +++ | |
|  | 3,75 |  | 1,08 | ± | 0,86 | + | | 0,79 | ++ | | 0,78 | ++ | |
|  | 7,50 |  | 1,22 | -- | 1,23 | -- | | 1,03 | ± | | 0,89 | + | |
|  | 15,00 |  | 1,87 | --- | 1,73 | --- | | 1,53 | --- | | 1,12 | - | |
|  | 30,00 |  | 1,66 | --- | 1,22 | -- | | 1,14 | - | | 0,92 | ± | |
|  |  |  |  |  |  |  | |  |  | |  |  | |
